# Intracerebroventricular BDNF infusion may reduce cerebral ischemia/reperfusion injury by promoting autophagy and suppressing apoptosis

**DOI:** 10.1111/jcmm.18246

**Published:** 2024-03-23

**Authors:** Umit Yilmaz, Kevser Tanbek, Semir Gul, Ahmet Koc, Mehmet Gul, Suleyman Sandal

**Affiliations:** ^1^ Department of Physiology, Faculty of Medicine Karabuk University Karabuk Turkey; ^2^ Department of Physiology, Faculty of Medicine Inonu University Malatya Turkey; ^3^ Department of Histology and Embryology, Faculty of Medicine Inonu University Malatya Turkey; ^4^ Department of Medical Biology and Genetics, Faculty of Medicine Inonu University Malatya Turkey

**Keywords:** adhesive removal test, autophagy, brain‐derived neurotrophic factor (BDNF), cerebral ischemia, neurological deficit score, rotarod test

## Abstract

Here, it was aimed to investigate the effects of intracerebroventricular (ICV) Brain Derived Neurotrophic Factor (BDNF) infusion for 7 days following cerebral ischemia (CI) on autophagy in neurons in the penumbra. Focal CI was created by the occlusion of the right middle cerebral artery. A total of 60 rats were used and divided into 4 groups as Control, Sham CI, CI and CI + BDNF. During the 7‐day reperfusion period, aCSF (vehicle) was infused to Sham CI and CI groups, and BDNF infusion was administered to the CI + BDNF group via an osmotic minipump. By the end of the 7th day of reperfusion, Beclin‐1, LC3, p62 and cleaved caspase‐3 protein levels in the penumbra area were evaluated using Western blot and immunofluorescence. BDNF treatment for 7 days reduced the infarct area after CI, induced the autophagic proteins Beclin‐1, LC3 and p62 and suppressed the apoptotic protein cleaved caspase‐3. Furthermore, rotarod and adhesive removal test times of BDNF treatment started to improve from the 4th day, and the neurological deficit score from the 5th day. ICV BDNF treatment following CI reduced the infarct area by inducing autophagic proteins Beclin‐1, LC3 and p62 and inhibiting the apoptotic caspase‐3 protein while its beneficial effects were apparent in neurological tests from the 4th day.

## 1 INTRODUCTION

Cerebral ischemia (CI) can result from a cerebral artery occlusion due to an embolus or clot that causes temporary or permanent blockage of blood flow to the brain.^1^ In an CI, several pathological processes such as disturbances in energy metabolism and oxidative stress, lead to necrotic and apoptotic cell death, leading to neuronal damage.^2^ In CI, the region where the decrease in cerebral blood flow is greatest and the cells are irreversibly damaged by necrosis within minutes is called the core, and the periphery of the core area is called the penumbra.^3^ The penumbra is the transition zone between the core and the non‐ischemic areas and is still supplied by small amounts of collateral arterial blood following ischemia. Penumbra has a lighter ischemic spread than the ischemic nucleus; therefore, cells here can regain their functions.^4^ There is a very short yet valuable time frame to recover damaged cells in the penumbra area, and with proper treatment some damaged cells can survive by autophagy. This makes autophagic cells in the ischemic penumbra a very important target for stroke therapy.^5^, ^6^

The fate of neurons after CI is determined by the balance between cell survival and death signals.^7^ Basal‐level autophagy is a necessary process for maintaining cellular homeostasis and adapting to adverse conditions.^8^ However, while inhibition of autophagy induces ischemic damage in the brain, excessively increased autophagy can increase cell death and thus cause neuronal death.^9^ That is, while appropriate autophagic activity is neuroprotective, excessive or insufficient autophagy often leads to cell death.^5^

Autophagy is a cellular process that recycles aggregate proteins, damaged organelles and certain pathogens via lysosomal degradation and plays an important role in cell survival by providing ischemic cells with additional energy sources and nutrients.^10^ The most important/critical step in autophagy is the formation of the vesicle, autophagosome and the selective recruitment of target organelles and proteins to this vesicle.^11^ Beclin‐1, Microtubule‐associated protein‐1 light chain 3 (LC3) and p62 proteins play a key role in this process. Beclin‐1 mediates the transport of other autophagy proteins to the pre‐autophagosomal membrane as the autophagosome is formed. LC3 has two forms, LC3‐I and LC3‐II; while LC3‐I is normally expressed in the cell, LC3‐II is expressed during autophagosome formation‐elongation, closure and fusion with the lysosome.^12^ p62 is an autophagy receptor that distinguishes damaged organelles from intact ones within the cell, allowing the autophagosome to recognise and selectively surround targets to be digested. The p62 protein itself is degraded in the lysosome along with the proteins it transports to the autophagosome.^13^

Brain‐Derived Neurotrophic Factor (BDNF) is a small dimeric protein encoded by the BDNF gene in humans that plays a role in the survival of neurons and the growth and differentiation of new neurons and synapses during the developmental period of the brain. BDNF is a member of the neurotrophins family, which plays a role in neuronal proliferation and migration, axonal and dendritic growth and branching, synapse formation and synaptic transmission. It can produce morphogenetic and chemotrophic effects and thus provide recovery after central nervous system injuries.^14^, ^15^

Brain‐Derived Neurotrophic Factor administered intravenously may play a protective role following CI^16^ however, systemic administration of BDNF has minimal therapeutic effect due to its poor penetration through the blood–brain barrier.^16^ In this study, it was aimed to reveal the effect of intracerebroventricular (ICV) BDNF administration for 7 days with osmotic minipumps following CI on autophagy. Moreover, it was also aimed to determine the impact of ICV BDNF infusion on neurological deficit scoring, rotarod and adhesive removal test during the reperfusion.

## 2 MATERIALS AND METHODS

### 2.1 Ethics statement and animal care

All animal experiments were conducted in accordance with the ethical guidelines designated by the Inonu University, Faculty of Medicine, Experimental Animal Ethics Committee and approved by the same committee (Protocol No: 2018/A‐46). The study was conducted with 60 male 220–280 g Sprague‐Dawley rats.^17^ The animals were kept in a temperature‐controlled (22 ± 1°C) environment with a 12/12‐h light/dark cycle and had ad libitum access to standard laboratory chow pellets and water.^18^ The study was conducted with the minimum number of animals, and maximum effort was spent to minimise any pain or discomfort.

### 2.2 Experimental design and groups

It was reported that systemic administration of BDNF has minimal therapeutic impact due to its poor penetration through the blood–brain barrier and short half‐life in serum.^16^ For this reason, ICV‐BDNF infusion was preferred in this study, as it was described in our previous study.^19^

Sixty rats were used in the investigation, with 15 rats in each group (six rats for Western blot, four rats for immunofluorescence analysis and five rats for infarct area calculation). After weighing each rat, those with similar body weights were randomised into four groups at random: Control, Sham CI, CI and CI + BDNF (*n* = 15).

**FIGURE 1:**
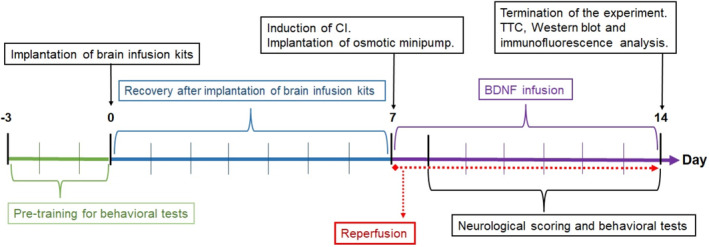
Experimental design.

The experimental design is summarised in Figure 1. The experimental design is as follows: The control group animals underwent a three‐day pre‐training for behavioural tests, but there was no subsequent brain infusion kit, CI, or osmotic minipump implantation. After 3 days of pre‐training for the behavioural test, the brain infusion kits were implanted into the right lateral ventricle of the animals in the Sham CI, CI and CI + BDNF groups (0‐day). After the procedure to implant the brain infusion kit, there was no administration for 7 days to allow for recovery period. After the 7‐day recovery period, sham surgical operations (all surgical operations except vessel occlusion) were performed in the Sham CI group, and a 90‐min transient focal CI was performed in the CI and CI + BDNF groups. A 7‐day reperfusion period was started after sham or 90‐min CI surgery (0‐day). Two hours after the start of reperfusion, osmotic minipumps were connected to the brain infusion kits of the Sham CI, CI and CI + BDNF groups and placed under the skin of the back (7th day). Subsequently, 1 μL/hour of artificial cerebrospinal fluid (aCSF; vehicle) was infused into the Sham CI and CI groups and BDNF (0.06 μg/1 μL/hour)^20^ was infused into the CI + BDNF group via osmotic minipumps for 7 days. Neurological deficit scoring and behavioural tests were initiated 24 h after the start of reperfusion (8th day). The scoring and tests were performed every day for 7 days during reperfusion (between the 8th and 14th days). On the seventh day of infusion (14th day), the final tests were performed, and the animals were decapitated. No animals died during the experiments, and each group still included 15 animals at the end of the experiments.

### 2.3 Implantation of brain infusion kits

The rats in the Sham CI, CI and CI + BDNF groups were anaesthetized with 70 mg/kg ketamine and 8 mg/kg xylazine intraperitoneally (0‐day). The anaesthetized rats were placed on the stereotaxic device, and the sculp was incised to access the skull. Then, the bregma point was used as the reference to determine the location of the right lateral ventricle based on the coordinates specified in the Paxinos‐Watson Stereotaxic Rat Brain Atlas (1.6 mm lateral, 0.8 mm posterior and 4.0 mm vertical from the bregma). The exact point was drilled without damaging the dura, and brain infusion kits (Alzet, cat: 8663, USA) were implanted in the right lateral ventricle. A closed‐ended cannula was connected to the brain infusion kit under the neck skin, and the animals were allowed to recover for 7 days to recover.^21^

### 2.4 Cerebral ischemia/reperfusion model induction

A 90‐min focal CI was initiated for the rats in the CI and CI + BDNF groups via the occlusion of the right middle cerebral artery (MCA) with the Koizumi intraluminal filament technique (7th day). The animals were anaesthetized, and their body temperature was kept constant at 36.5–37°C during the experiment. Initially, the right common carotid artery (CCA), carotid bifurcation, external carotid artery (ECA) and internal carotid artery (ICA) were dissected from the surrounding tissues for exposure. The ECA was ligated distally and the CCA was ligated proximally, and a microvascular clamp was temporarily placed on the ICA. The right CCA was incised, and a 4/0 nylon filament, the tip of which was coated with silicone resin, was inserted into the vein through this incision and advanced towards the ICA. Then, the clamp placed on the ICA was removed and the nylon filament was advanced towards the MCA until a slight resistance was felt (approximately 18–20 mm). Ischemia was induced by keeping the nylon filament at position for 90 min, and then the filament was removed to induce reperfusion. In the Sham CI group, the same surgical procedure was performed without inserting a filament into the vessel. After the surgery, incision area was sutured, and animals were placed back into their cages.^22^

### 2.5 Implantation of osmotic minipumps

Osmotic minipumps operate on the basis of the osmotic pressure difference between a compartment within the pump, called the osmotic layer, and the tissue environment in which the pump is implanted. When water from the tissue environment in which the pump is implanted enters the osmotic layer of the pump, it compresses the flexible reservoir and removes the solution inside the pump from the pump at a controlled, predetermined rate. The osmotic minipumps capable to infuse 1 μL/h for 7 days were employed in the study (Alzet, Cat: 2001, USA). Osmotic minipumps were filled with BDNF (0.06 μg/h)^20^ in the CI + BDNF group and with the solvent aCSF in the Sham CI and CI groups. On the 2nd hour of post‐CI reperfusion, the animals were anaesthetized again with 60 mg/kg ketamine and 5 mg/kg xylazine intraperitoneally (7th day). The tip of the brain infusion kit cannula was connected to the osmotic minipump to initiate BDNF and aCSF infusions. Then, osmotic minipumps were implanted under the back skin, the incision area was sutured, and the animals were placed back into their cages.^21^

### 2.6 Behavioural tests

#### 2.6.1 Neurological score

Neurological deficit scoring was conducted on all animals at the same time every day for 7 days based on the modified Bederson scoring method 24 h after the reperfusion was initiated (8th day). 0 = Normal neurological examination, 1 = flexion of the contralateral forelimb when lifted by the tail, 2 = flexion of the contralateral forelimb and decreased resistance to lateral push, 3 = circling in the contralateral direction while walking, 4 = turning or seizure when lifted by the tail, 5 = inability to walk spontaneously.^23^

#### 2.6.2 Rotarod test

The rotarod test was applied to the animals after the stroke to evaluate their resistance and their balanced and coordinated movements. Before the CI, all rats were pre‐trained for 3 days.^24^ Tests were conducted on all animals at the same time for 7 days, 24 h after the reperfusion was initiated (8th day). The speed of the rotarod device was increased from 4 rpm to 40 rpm within 5 min, and the time the rats could stay on the rod was measured. The experiment was repeated five times for each animal, and the mean trial finding was compared for each day.^25^

#### 2.6.3 Adhesive removal test

The adhesive removal test was used to determine the sensorimotor dysfunction (somatosensory neglect) and motor asymmetry after the stroke. In this test, the animal is expected to remove small adhesive labels of equal size (1 × 1 cm^2^) placed on the distal radial surface of both forepaws. All animals underwent a 3‐day acclimation before the CI. The adhesive removal test was conducted with all animals every day for 7 days, 24 h after the reperfusion was initiated (8th day). The time it took for the animals to remove the adhesive tapes from each paw was recorded. All mean trial findings were compared every day for each animal.^26^

#### 2.6.4 Measurement of infarct area

The rats were decapitated after the 7‐day reperfusion, and the brains were removed and frozen on dry ice. The brain was sliced at 2 mm intervals from the +4 mm anterior and −6 mm posterior of the bregma. Then, the sections were incubated in 1% TTC (2,3,5‐triphenyltetrazolium chloride; Sigma‐Aldrich, USA) for 10 min at 37°C in the dark. After TTC staining, the sections were fixed with 4% formalin. The infarct and non‐infarct area boundaries in the stained sections were determined with the Image J software. The infarct areas (%) were calculated with the following formula: (area of the contralateral hemisphere − non‐infarct area of the ipsilateral hemisphere)/(non‐infarct area of the ipsilateral hemisphere) × 100.^27^, ^28^

#### 2.6.5 Dissection of ischemic penumbra area

**FIGURE 2:**
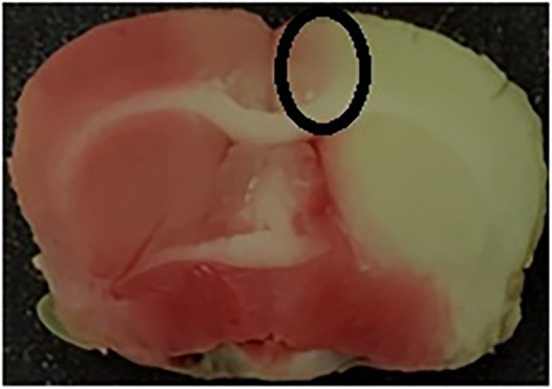
Penumbra area used in Western blot and immunofluorescence analyses.

The ischemic penumbra area in the cortex is presented in Figure 2. Initially, the brain was divided into three sections, starting 3 mm from the anterior end of the frontal lobe. The anterior and posterior sections were 3 mm thick. The middle section was 4 mm thick and was incised 2 mm longitudinally from the midline in the ischemic hemisphere. To separate the core region from the penumbra, a transverse cut was performed at the 2 o'clock position to obtain the penumbra in the cortex. Western blot and immunofluorescence analysis were performed using penumbra area in the cortex.^29^

#### 2.6.6 Western blot

For the Western blot analyses of the study, brain tissues from 6 rats were randomly collected from each group (4 groups: Control, Sham CI, CI and CI + BDNF) (24 brain tissues in total). The ischemic penumbra tissues obtained from the cortex of the six rats in each group were individually homogenized and lysed with RIPA Lysis Buffer (ab156034, Abcam, UK) supplemented with protease and phosphatase inhibitor cocktail (ab201111, Abcam, UK). Total protein concentration in the brain tissue protein mix was determined with the BCA kit (ab102536, Abcam, UK). Equal amounts of the protein (40 μg) were separated by SDS‐PAGE and transferred to the PVDF membrane. The PVDF membrane was blocked with 5% skim milk powder prepared with the TBS‐T for 1 h at room temperature. PVDF membranes were incubated with primary antibodies (Beclin‐1 (2 μg/mL, ab62557, Abcam, UK), LC3‐I/II (1:1), LC3‐I/II (1:500, ab128025, Abcam, UK), p62 (2 μg/mL, ab91526, Abcam, UK) and cleaved caspase‐3 (1:500, ab49822, Abcam, UK)/β‐actin (1:1000, ab8226, Abcam, UK)). After incubation, the membrane was washed three times for 15 min with TBS‐T. After the membrane was incubated with the secondary antibody (anti‐rabbit, 1:2000, ab205718, Abcam, UK) for 1 h at room temperature, it was washed three times for 15 min with TBS‐T to remove the secondary antibody that did not bind to the primary antibody. Then, the membrane was treated with enhanced chemiluminescence (ECL) solution (Thermo Scientific, USA, cat. no: 32106) and imaged with the UVP Syngene G:BOX Chemi‐XRQ Gel Documentation System. Protein bands were calculated with GeneTools software (Version 4.03.05, Sygene, Cambridge, UK).^22^, ^30^ Finally, to include representative Western blot images of the groups in the article, one rat brain tissue from each group was selected and run in gel for Beclin‐1, LC3, p62, caspase‐3 and β‐actin genes. Therefore, in the article, there is the same β‐actin gel image below the gel images of Beclin‐1, LC3, p62 and caspase‐3 genes.

#### 2.6.7 Immunofluorescence

Three equal 8‐μm coronal sections were obtained from the bregma level (0.0 mm) of the brain tissues of four rats in each group with a freezer microtome (Leica CM1850, Germany), and the sections were adhered to positively charged slides. The sections were kept in acetone at −20°C for 15 min for fixation, and then the sections were washed twice with PBS buffer at +4°C in 5‐min intervals. Then, the sections were incubated with 1% BSA for 30 min. Then, primary antibodies Beclin‐1 (20 μg/mL, ab62557, Abcam, UK), LC3 (2 μg/mL, ab128025, Abcam, UK), p62 (20 μg/mL, ab91526, Abcam, UK) were placed on the sections, and cleaved caspase‐3 (1:500, ab49822, Abcam, UK) was added, and they were incubated at +4°C overnight. The sections were washed three times with PBS buffer at 5‐min intervals and incubated with the secondary antibody Alexa Fluor® 488 (1:500, ab150077, Abcam, UK) for 1 h in the dark at room temperature. After the sections were washed 3 times with PBS buffer at 5‐min intervals, they were treated with the nuclear dye DAPI (Diamidine‐2‐Phenylindole Dihydrochloride) (ab104139, Abcam, UK) for 5 min. After the sections dried on the slide in the dark, they were covered with Gel/Mount fluid protection, a coverslip, and stored at −20°C until the analysis. The sections were digitally photographed with a fluorescent microscope (NIS‐Elements Documentation 5.02 Nikon Instruments Inc., Melville, NY). Image J Cell Counter plugin was employed to mark and count Beclin‐1, LC3, p62 and cleaved caspase‐3 positive neurons in the penumbra. Once the positive neurons were labelled, Image J was employed to overlay the images to measure colocalization. The selected regions were counted in the peri‐infarct area of three cortical series for each rat. All counts were conducted by two academicians who were blind to the study groups.^22^, ^30^

### 2.7 Statistical analysis

The study data were analysed with the IBM SPSS software version 25.0 for Windows. The normal distribution of the data was analysed with the Shapiro–Wilk test. Since the neurological deficit scores, rotarod test and adhesive removal test data did not exhibit normal distribution, the differences between the groups were analysed with the Kruskal–Wallis *H* test. Then, Mann–Whitney *U* test with Bonferroni correction was employed for inter‐group comparisons. Friedman test was used for the intra‐group (day‐to‐day) comparisons. Kruskal–Wallis *H* test was employed to analyse Western blot and immunofluorescence findings. When there were significant differences between the groups, multiple/pairwise comparisons were conducted with the Mann–Whitney *U* test with Bonferroni correction. *p* < 0.05 was considered statistically significant.

## 3 RESULTS

### 3.1 BDNF treatment could improve neurological score

**FIGURE 3:**
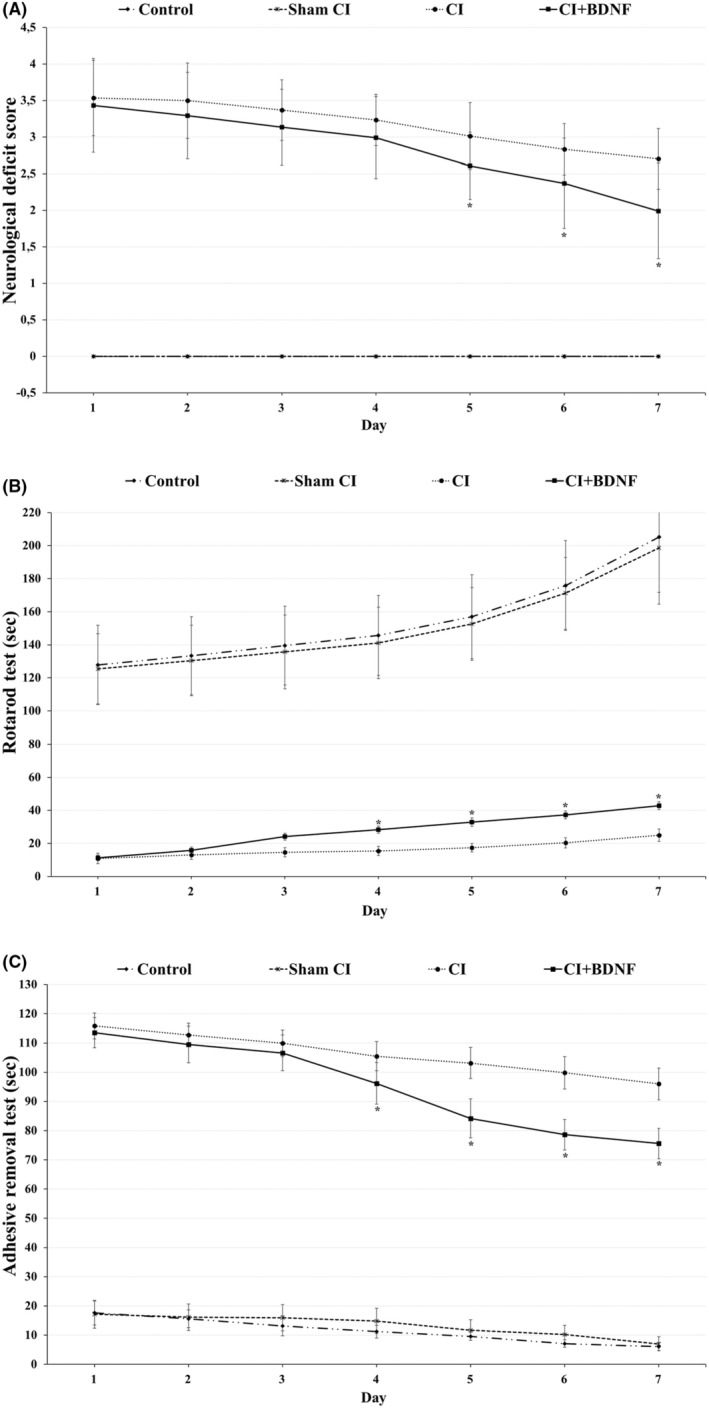
(A) ICV‐BDNF treatment decreased neurological score. During the 7‐day reperfusion period (between the 8th and 14th days), BDNF treatment improved neurological deficit score on the 5th, 6th, and 7th days (*p* < 0.05; dF value is 3). The symbol (*) in the graph means that the CI + BDNF group is statistically different from the CI group (*n* = 15). (B) ICV‐BDNF treatment increased rotarod test duration. During the 7‐day reperfusion period (between the 8th and 14th days), BDNF treatment improved motor coordination and balance on the 4th, 5th, 6th and 7th days (*p* < 0.05; dF value is 3). The symbol (*) in the graph means that the CI + BDNF group is statistically different from the CI group (*n* = 15). (C) ICV‐BDNF treatment reduced the adhesive removal test time. During the 7‐day reperfusion period (between the 8th and 14th days), BDNF treatment recovered sensorimotor dysfunction (somatosensory neglect) and motor asymmetry on days 4, 5, 6 and 7 (*p* < 0.05; dF value is 3). The symbol (*) in the graph means that the CI + BDNF group is statistically different from the CI group (*n* = 15).

The neurological deficit scoring was conducted to determine motor coordination and sensorimotor dysfunctions in rats during the post‐CI 7‐day reperfusion. The comparison of the groups based on neurological deficit scores demonstrated that the neurological deficit scores of the Control and Sham CI groups were significantly lower when compared to the ischemia and BDNF groups between the 1st and 7th days (*p* < 0.05). ICV BDNF administration significantly reduced the neurological deficit score, which increased after the CI on the 5th, 6th and 7th days (*p* < 0.05). Furthermore, the daily comparison of the ischemia and treatment groups revealed that the neurological deficit score of the groups decreased significantly when compared to the previous day (*p* < 0.05; Figure 3).

### 3.2 BDNF treatment could improve motor coordination and balance

The rotarod test was conducted to determine the motor coordination, balance and grip strength of the rats during post‐CI reperfusion. The inter‐group comparison of the rotarod test times demonstrated that they were significantly higher in the control and Sham CI groups when compared to ischemia and BDNF groups between the 1st and 7th days (*p* < 0.05). ICV BDNF administration significantly increased the rotarod test time, which decreased after CI, on the 4th, 5th, 6th and 7th days (*p* < 0.05). Furthermore, the daily comparison of all groups revealed that rotarod duration increased significantly when compared to the previous day (*p* < 0.05) (Figure 3B).

### 3.3 BDNF treatment could recover sensorimotor dysfunction (somatosensory neglect) and motor asymmetry

The adhesive removal test that measured sensorimotor dysfunction (somatosensory neglect) and motor asymmetry was conducted during the post‐CI reperfusion. The inter‐group comparison of the adhesive removal test times demonstrated that it was significantly lower in the Control and Sham CI groups when compared to the ischemia and BDNF treatment groups between the 1st and 7th days (*p* < 0.05). ICV BDNF treatment significantly reduced the increased post‐CI adhesive removal time on the 4th, 5th, 6th and 7th days (*p* < 0.05). Furthermore, the daily comparison of all groups revealed that the adhesive removal time decreased significantly in all groups when compared to the previous day (*p* < 0.05; Figure 3C).

### 3.4 BDNF treatment decreased the post‐CI infarct area

**FIGURE 4:**
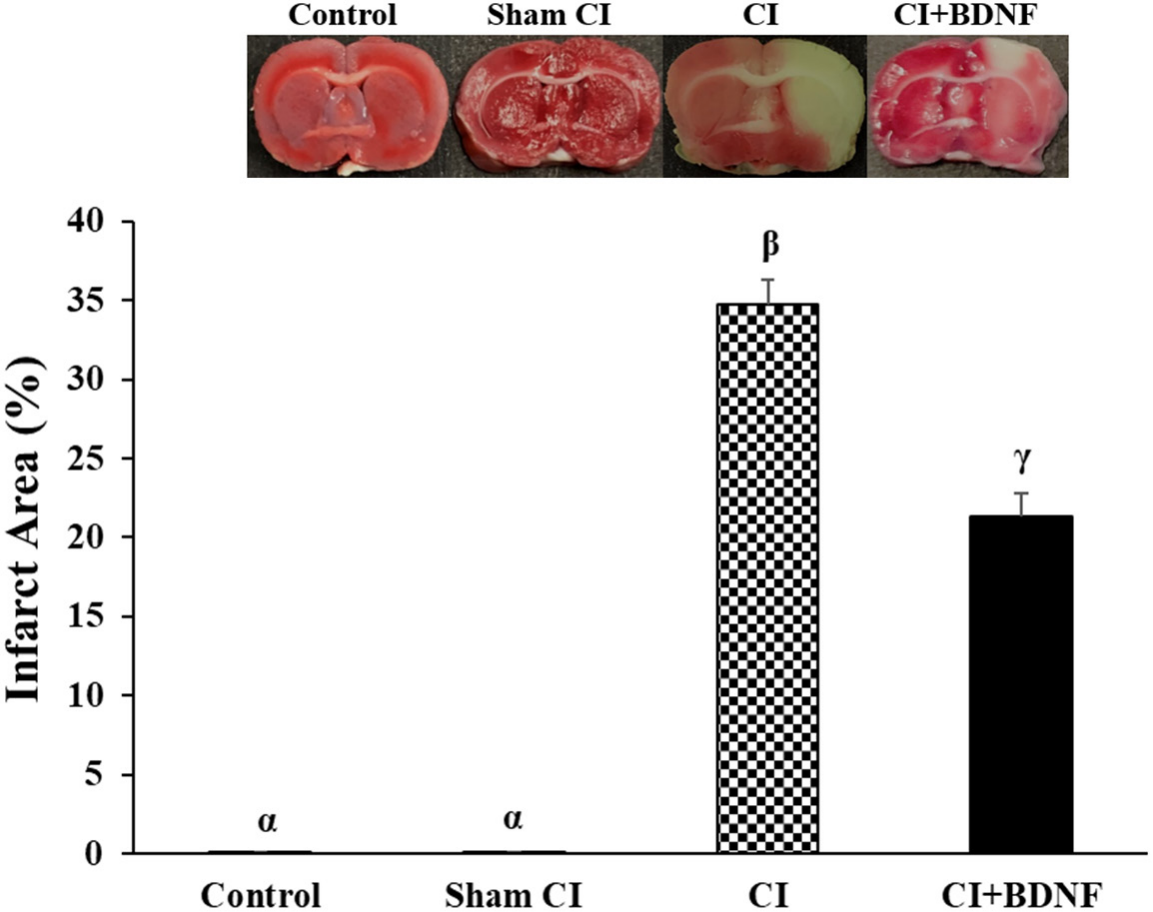
ICV‐BDNF treatment decreased the infarct area after CI. The groups marked with different symbols were statistically different from each other (*p* < 0.05).

The rats in all groups were decapitated 7 days after the CI, and infarct areas were determined with TTC staining. No infarct area was determined in the control and sham group rat brains. The comparison of the groups based on the post‐CI infarct area demonstrated that it was significantly larger in the ischemia (34.7%) and BDNF (21.3%) groups when compared to the Control and Sham CI groups (*p* < 0.05). However, ICV BDNF administration significantly reduced the post‐ischemia infarct area (*p* < 0.05; Figure 4).

### 3.5 BDNF treatment could induce autophagy in the area around the ischemic core

To determine whether BDNF, a protective agent against post‐CI and reperfusion damages, induced autophagy to rescue the damaged neuronal cells after CI, we examined the protein levels associated with autophagy (Beclin‐1, LC3‐II and p62) with Western blot and immunofluorescence methods. The simultaneous increase in Beclin‐1 and LC3‐II proteins and the decrease in p62 protein indicated autophagy activation.

**FIGURE 5:**
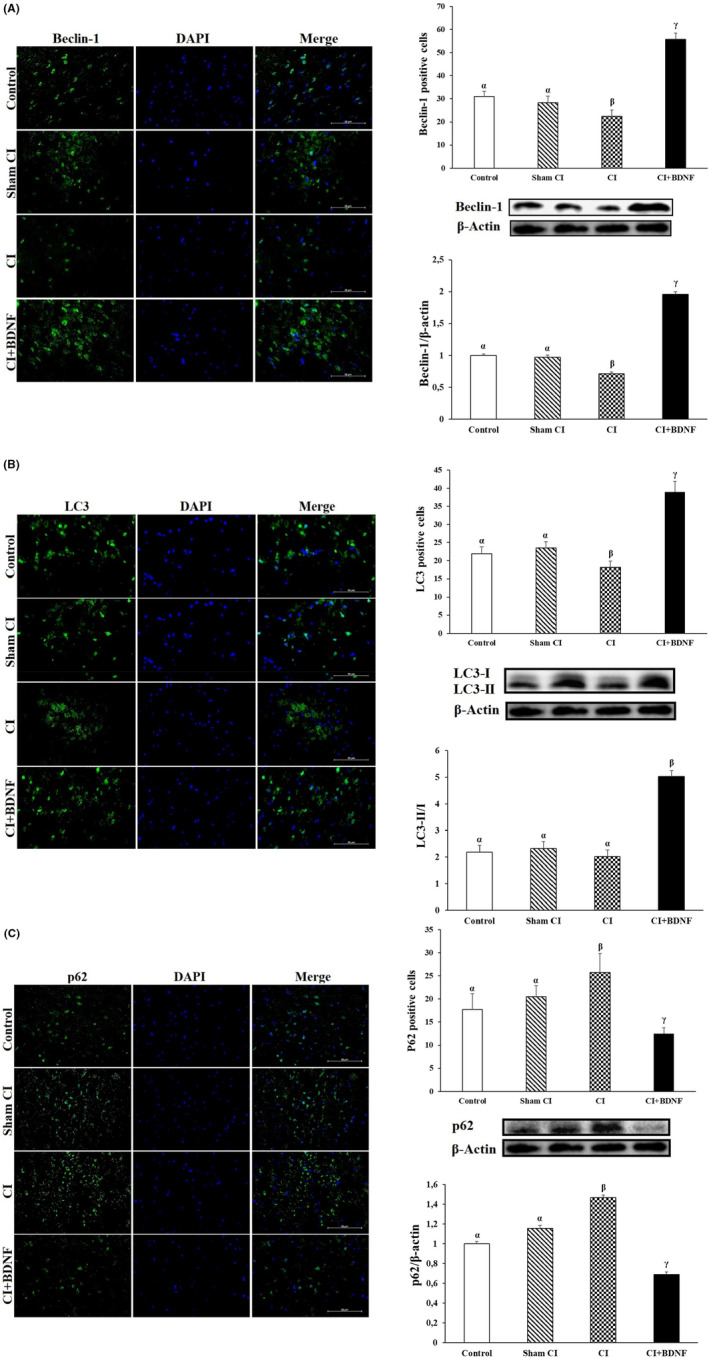
(A) ICV‐BDNF treatment induced Beclin‐1 protein levels and positive cells in the penumbra area after CI. The groups marked with different symbols were statistically different from each other (*p* < 0.05). There is the same β‐actin gel image below the gel images of Beclin‐1, LC3, p62, caspase‐3 genes. (Beclin‐1 (green) with the nuclei counterstained by DAPI (blue). Scale bar = 50 μm). (B) ICV BDNF treatment induced LC3‐II/I protein and LC3 positive cells after CI. The groups marked with different symbols were statistically different from each other (*p* < 0.05). There is the same β‐actin gel image below the gel images of Beclin‐1, LC3, p62, caspase‐3 genes. (LC3 (green) with the nuclei counterstained by DAPI (blue). Scale bar = 50 μm). (C) ICV BDNF treatment reduced p62 protein levels and positive cells after CI. The groups marked with different symbols were statistically different from each other (*p* < 0.05). There is the same β‐actin gel image below the gel images of Beclin‐1, LC3, p62, caspase‐3 genes. (p62 (green) with the nuclei counterstained by DAPI (blue). Scale bar = 50 μm).

Ninety minutes after CI, it was determined that the Beclin‐1 protein level and the number of Beclin‐1 positive cells in the ischemia group decreased in the penumbra area when compared to the Control and Sham CI groups (*p* < 0.05). However, during the post‐CI reperfusion, ICV BDNF treatment increased the Beclin‐1 protein level and the number of positive cells in the penumbra (*p* < 0.05; Figure 5).

LC3‐II/I conversion was also determined to quantify autophagosomes in the penumbra. The comparison of the groups based on the post‐CI LC3‐II/I protein ratio in the penumbra revealed that the LC3‐II/I ratio decreased in the CI group when compared to the Control; however, the difference was not significant (*p* > 0.05). However, the LC3‐II/I protein ratio significantly decreased in the CI group when compared to the Sham CI group (*p* < 0.05). ICV BDNF treatment significantly increased the post‐CI LC3‐II/I protein ratio in the penumbra (*p* < 0.05). The inter‐group comparison of the penumbra areas based on the number of LC3 positive cells demonstrated that the number of post‐CI LC3 positive cells was lower in the penumbra when compared to the Control and Sham CI groups; however, ICV BDNF treatment increased the number of LC3 positive cells (*p* < 0.05; Figure 5B).

It was determined that the post‐CI autophagic protein p62 level and the number of positive cells increased in the ischemia group when compared to the Control and Sham CI groups (*p* < 0.05). However, the post‐CI BDNF treatment reduced p62 protein level and the number of p62 positive cells in the area around the ischemic core (*p* < 0.05; Figure 5C).

These findings demonstrated that autophagic proteins were inhibited in the penumbra after CI/R injury; however, ICV BDNF treatment could have a neuroprotective effect by inducing autophagy.

### 3.6 BDNF treatment inhibited apoptotic cleaved caspase‐3 protein

**FIGURE 6:**
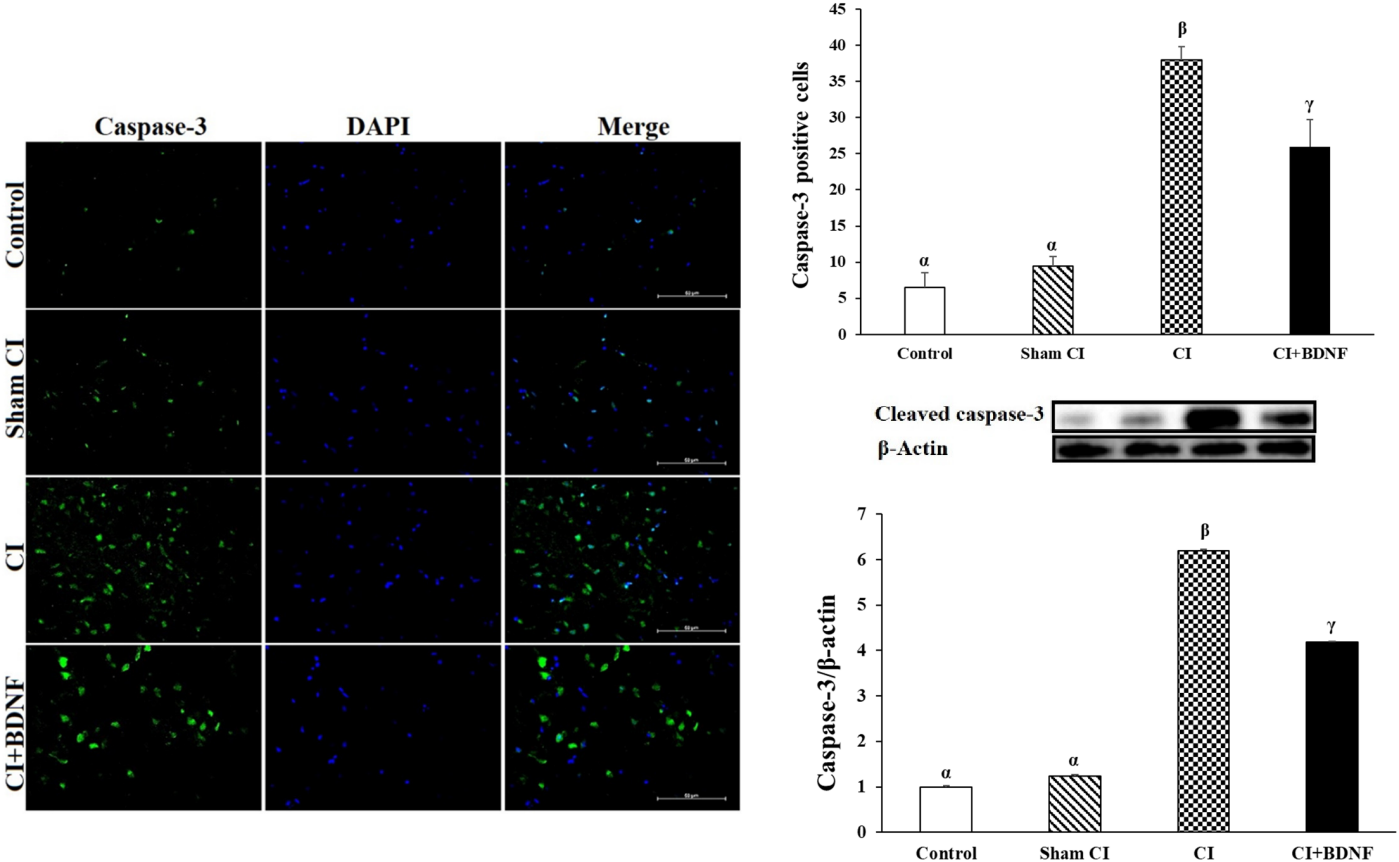
IC BDNF treatment suppressed apoptotic protein cleaved caspase‐3 protein levels and positive cells after CI. The groups marked with different symbols were statistically different from each other (*p* < 0.05). There is the same β‐actin gel image below the gel images of Beclin‐1, LC3, p62, caspase‐3 genes. (Cleaved caspase‐3 (green) with the nuclei counterstained by DAPI (blue). Scale bar = 50 μm).

Caspase 3 is often activated to catalyse the cleavage of certain downstream molecules, which ultimately lead to DNA fragmentation and programmed cell death, known as apoptosis.^31^ Post‐CI cleaved caspase‐3 protein levels and positive cell count were high in both ischemia and BDNF treatment groups in the penumbra (*p* < 0.05). However, ICV BDNF treatment reduced the cleaved caspase‐3 protein level and the number of positive cells, which increased after ischemia in the penumbra (*p* < 0.05; Figure 6). In conclusion, these findings suggested that ICV BDNF treatment could inhibit caspase‐3 in the penumbra and prevent apoptosis.

## 4 DISCUSSION

The BDNF has been shown to mediate oligodendrogenesis and remyelination and play a key role in angiogenesis, synaptogenesis and functional recovery after stroke.^16^ Studies conducted to elucidate the pathophysiology of stroke suggested that neuronal cell death occurred due to apoptosis after ischemic brain injury.^32^, ^33^ However, focal CI does not only impair autophagy in neurons, glial cells and brain microvascular cells but also plays the “good cop/bad cop” at different times after ischemic injury.^34^

There are no previous studies where a focal CI model was developed to investigate the effects of ICV BDNF infusion on apoptosis and autophagy. The present study findings suggested that a 7‐day‐long ICV BNDF infusion after 90 min of focal CI reduced the apoptotic cleaved caspase‐3 protein levels and increased the autophagic Beclin‐1, LC3 and p62 protein levels. It could also be suggested that BDNF infusion improved neurological deficit score, rotarod and adhesive removal test results on the seventh day.

Previous studies reported that BDNF transfection prevented cellular death in CI by specifically upregulating the TrkB receptor in cortical neurons in the penumbra.^35^ BDNF was administered with several methods after CI to investigate its effects. In a study that investigated the effects of intranasal BDNF administration in CI, it was reported that a single dose of post‐CI intranasal BDNF administration could protect the brain against ischemic damage by modulating local inflammation; however, BDNF it did not have a significant impact on the infarct area.^36^ In another study, it was demonstrated that intranasal BDNF administration via small extracellular vesicles stimulated neuroprotective genes and inhibited inflammatory genes in mice with focal CI. Furthermore, BDNF was reported to reduce post‐CI infarct volume and improve neurological score and rotarod duration.^37^ The findings of both studies demonstrated that intranasal BDNF administration via small extracellular vesicles could be more beneficial in the protection of the brain against ischemic damage. Another application method where the effects of post‐CI BNDF administration were investigated was intravenous administration. It was reported that post‐CI intravenous BDNF administration improved the neurological deficit score and adhesive removal time of the rats on day 7.^38^ Kalinichenko et al.^39^ suggested that intravenous BNDF infusion after focal CI reduced the infarct area and regulated ischemic tolerance the mechanisms in neurons. Schabitz et al.^40^ reported that intravenous BDNF administration after stroke stimulated neurogenesis and improved neurological deficit score, rotarod and adhesive removal times. Another study reported that a single dose of post‐CI intravenous BDNF treatment reduced the infarct area and improved the neurological deficit score after 24 h. It was also reported that BDNF treatment decreased Bax protein and increased Bcl‐2 protein levels, providing neuroprotection by inhibiting apoptosis.^41^ Zhang et al. reported that post‐CI intravenous BDNF administration had no effect. However, when they formulated BDNF to pass the blood–brain barrier, BDNF reduced the post‐CI infarct area and increased the rotarod test duration.^42^ Zhang et al.'s report contradicted previous studies in the sense that intravenous BDNF administration without binding to any agent could not cross the blood–brain barrier and have a neuroprotective effect in CI.

It was reported that post‐CI ICV BDNF administration of for 7 days globally reduced neuronal death,^20^ and 14 days of administration improved long‐term memory and cognitive functions.^43^ It was demonstrated that single‐dose ICV BDNF administration before CI improved motor‐sensory, sensorimotor and vestibular motor functions but did not reduce the infarct volume.^44^ In a study by Plowman et al.,^45^ where antisense BDNF oligonucleotide was administered with osmotic minipumps after focal CI, it was reported that BDNF reduced the infarct area, improved motor functions and contributed to the recovery of the skills during rehabilitation. The design of the studies conducted by Schabitz et al.,^46^ Yanamoto et al.,^47^ and Yamashita et al.^48^ was similar to the design of our study. In these three studies, ICV BDNF was administered with osmotic minipumps after CI. All three studies reported that ICV BDNF administration with osmotic minipumps reduced the post‐CI infarct area and exhibited neuroprotective properties. Also, Schabitz et al.^46^ demonstrated that ICV BDNF administration improved the neurological deficit score. No studies attempted to develop a focal CI model to investigate the effects of BDNF on molecular autophagy. However, a few studies have investigated the effect of BDNF on apoptosis in hypoxic–ischemic (HI) models. Han et al.^49^ reported that a single ICV BDNF dose on the 7th postnatal day mediated neuroprotection via the activation of the ERK pathway in neonatal HI, a cerebral palsy model, and a single ICV BDNF dose before neonatal HI led to caspase‐3 activation. They reported that it blocked neuronal loss by preventing apoptosis.^50^ It was reported that ICV mesenchymal stem cell transplantation in a HI‐induced rat brain injury model contributed to neuroprotection by increasing BDNF levels and autophagy.^51^ It was also reported that BDNF administration in HI neuronal cell culture increased LC3‐II levels and exhibited neuroprotective properties via autophagy.^52^

In conclusion, it could be suggested that post‐CI ICV BNDF infusion prevented neuronal cell loss in the penumbra by inhibiting the apoptotic protein caspase‐3 and inducing the autophagic Beclin‐1, LC3 and p62 proteins It also recovered neurons with damaged organelles and proteins in the penumbra via autophagy, reducing the infarct area. Furthermore, the recovery of neurons in the penumbra led to the improvement in neurological deficit scoring, motor coordination (rotarod test) and sensory functions (adhesive removal test) in the BDNF‐treatment group.

## 5 CONCLUSION

It was shown that stimulation of autophagy was more beneficial when compared to inhibition in the prevention of neuronal damage and stimulation of autophagy ensured post‐CI neuronal survival.^53^ In conclusion, the present study findings demonstrated that ICV BDNF treatment could ensure neuronal survival, inhibit apoptosis and reduce the infarct area by upregulation of autophagic proteins after CI.

## AUTHOR CONTRIBUTIONS

**Umit Yilmaz:** Conceptualization (equal); data curation (equal); formal analysis (equal); funding acquisition (equal); investigation (equal); methodology (equal); project administration (equal); resources (equal); writing – original draft (equal); writing – review and editing (equal). **Kevser Tanbek:** Data curation (equal); formal analysis (equal); methodology (equal). **Semir Gul:** Formal analysis (equal); methodology (equal). **Ahmet Koc:** Formal analysis (equal); methodology (equal). **Mehmet Gul:** Formal analysis (equal); methodology (equal). **Suleyman Sandal:** Conceptualization (equal); writing – original draft (equal); writing – review and editing (equal).

## FUNDING INFORMATION

The present work was supported by the Research Fund of Inonu University [Project Number: TSA‐1507].

## CONFLICT OF INTEREST STATEMENT

The authors declare that they have no conflict of interest.

## Data Availability

Data available on request from the authors.
